# Novel bioglasses for bone tissue repair and regeneration: Effect of glass design on sintering ability, ion release and biocompatibility

**DOI:** 10.1016/j.matdes.2017.05.037

**Published:** 2017-09-05

**Authors:** Elena Mancuso, Oana A. Bretcanu, Martyn Marshall, Mark A. Birch, Andrew W. McCaskie, Kenneth W. Dalgarno

**Affiliations:** a School of Mechanical and Systems Engineering, Newcastle University, UK; b School of Mechanical Engineering, University of Leeds, UK; c Glass Technology Services Ltd, Sheffield, UK; d Division of Trauma and Orthopaedic Surgery, University of Cambridge, UK

**Keywords:** Glass design, Sintering ability, Ion release, Biocompatibility, Bone substitutes

## Abstract

Eight novel silicate, phosphate and borate glass compositions (coded as NCLx, where x = 1 to 8), containing different oxides (*i.e.* MgO, MnO_2_, Al_2_O_3_, CaF_2_, Fe_2_O_3_, ZnO, CuO, Cr_2_O_3_) were designed and evaluated alongside apatite-wollastonite (used as comparison material), as potential biomaterials for bone tissue repair and regeneration. Glass frits of all the formulations were processed to have particle sizes under 53 μm, with their morphology and dimensions subsequently investigated by scanning electron microscopy (SEM). In order to establish the nature of the raw glass powders, X-ray diffraction (XRD) analysis was also performed. The sintering ability of the novel materials was determined by using hot stage microscopy (HSM). Ionic release potential was assessed by inductively coupled plasma optical emission spectroscopy (ICP-OES). Finally, the cytotoxic effect of the novel glass powders was evaluated for different glass concentrations *via* a colorimetric assay, on which basis three formulations are considered promising biomaterials.

## Highlights

• Eight novel bioglass compositions based on silicate, phosphate and borate network formers have been investigated;
• The glass forming ability of the formulations was composition dependent;
• HSM proved an effective method to obtain accurate information about the thermal behaviour of the novel formulations;
• Three formulations are considered to show promise for future application as musculoskeletal biomaterials.

## Graphical abstract

**Figure:**
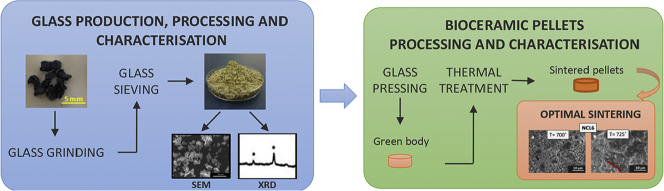

## 1 Introduction

The first reported use of a glass intended for bone tissue repair dates back to 1969, when Professor L. Hench proposed a composition in the Na_2_O-CaO-SiO_2_-P_2_O_5_ system, designated as bioglass 45S5 [1], [2], commercially known as Bioglass®.

Although Bioglass® proved to be an excellent material, considered for long time the gold standard bone substitute, it suffers from several drawbacks. Specifically, the main difficulties are related to the material processing in form of 3D porous scaffolds, due to the limited ability of this glass in sintering [3]. Additionally, other weaknesses include: its slow degradation kinetic with the consequent difficulties to match the formation rate of new tissue, and the abrupt pH variations of the biological microenvironment, due to the increase in the concentration of ions such as Na^+^ and Ca^2 +^, especially in the short term when the release is faster [4], [5], [6].

Worldwide many researchers have used the SiO_2_-Na_2_O-CaO-P_2_O_5_ system as a template for developing new silica-based compositions [7]. Subsequently, many formulations in the phosphate and borate-based system have been also designed to overcome the Bioglass® and silicate-based glass limitations [8], [9], [10], [11], and thus to meet the set of requirements that are both crucial and necessary for optimised tissue-engineered substitutes [12].

The possibility to tailor glass properties by doping the main composition with network modifiers and/or intermediate oxides [13], [14], [15], [16], [17], [18], [19], [20] offers significant potential for this class of biomaterials. In addition to promoting bone bonding, the release of soluble ions (*i.e.* Si, Ca, P and Na) from these glasses have been demonstrated to promote cell proliferation, differentiation and activate gene expression [20], [21], [22], [23], [24]. Furthermore, it has been also revealed that even slight changes in the glass main formulation can substantially affect the material behaviour, particularly the physico-chemical and mechanical properties, dissolution rate, bioactivity and bioresorbability [5], [16], [18], [25], [26], [27].

However, there are still several criticisms related to the clinical use of this class of biomaterials in bone repair [12]. Firstly, whether or not glass dissolution products have a positive effect on adult stem cells is still an open debate [28]. Secondly, they have often proved inadequate when used in load-bearing bone defects, due to their low tensile strength and fracture toughness [29]. Ultimately, there are no large-scale porous bioactive glasses on the market, thus their commercial success as bone scaffolds is limited [6], [12].

The aim of this work was the development and characterisation of eight novel silicate, phosphate and borate glass formulations (coded as NCLx, where x = 1 to 8), containing different oxides and in diverse molar percentages as promising biomaterials for the repair and regeneration of bone tissue.

## 2 Materials and methods

### 2.1 Development of novel glass formulations

Based on the current state of the art, the eight bioceramic formulations were developed using: silicon dioxide, phosphorous pentoxide and boron trioxide as network formers due to their promising bioactive potential [1], [30], [31], distinctive resorbable properties [32], [33], and customable degradation rate [5], [34], along with a range of different doping agents (*i.e.* MgO, MnO_2_, Al_2_O_3_, CaF_2_, Fe_2_O_3_, ZnO, CuO, Cr_2_O_3_), which were used to tailor the properties of the main composition [35], [36], [37], [38], [39], [40], [41]. The rationale and innovative characteristics of the novel materials are reported in Table 1. Additionally, considering the excellent biocompatibility either *in vitro* and *in vivo* of apatite wollastonite (AW) [42], [43], [44], and the fact that it has been adopted for a broad range of medical applications, either in the form of powder, porous structures or bulk material [45], [46], AW glass-ceramic was used as comparison material in this study.

**Table 1:** Rationale of the novel glass compositions.

CODE	MAIN NETWORK FORMER	AIM
NCL1	SiO_2_	To develop a material with osteogenic properties, mainly determined by the presence of a high amount of silica.
NCL2	SiO_2_	To develop a load-bearing material with osteogenic properties and tailored degradation rate.
NCL3	B_2_O_3_	To develop a material with improved degradation rate and appropriate level of bioactivity as well as mechanical properties
NCL4	B_2_O_3_	To develop a material with tailored degradation rate and osteogenic effects.
NCL5	P_2_O_5_	To develop a resorbable glass with controlled degradation rate.
NCL6	P_2_O_5_	To develop a resorbable glass with controlled degradation rate, and improved mechanical strength
NCL7	SiO_2_	To develop a material with antibacterial properties, mainly determined by the presence of silver oxide, and a good level of bioactivity.
NCL8	SiO_2_	To develop a material with osteogenic properties and tailored degradation rate for non-load bearing applications.

### 2.2 Glass production and processing

The novel glasses were produced and supplied by Glass Technology Service (GTS) Ltd. (Sheffield, UK) along with AW. Briefly, the individual components (see Table 2) of each formulation were weighed out and then mixed together to obtain a uniform blend, which was subsequently melted in platinum crucibles at temperatures up to 1500 °C. The individual melts of glass were cast as solid blocks and then thermally shocked in de-ionised water to produce the precursor materials, known as frits.

**Table 2:** Composition of the novel glass formulations.

CODE	GLASS COMPOSITION (wt%)
NCL1	30.44SiO_2_–9P_2_O_5_–6.29Na_2_O–7.10CaO–9.54K_2_O–5.56MgO–5.52MnO_2_–5.16ZnO–13.14SrO–1.01CuO–5.91Bi_2_O_3_–2.02TeO_2_–2.31V_2_O_5_
NCL2	36.90SiO_2_–9.70P_2_O_5_–1.90B_2_O_3_–3.39Na_2_O–11.48CaO–3.85K_2_O–4.41MgO–2.38MnO_2_–6.97Al_2_O_3_–2.13CaF_2_–10.92Fe_2_O_3_–0.41Li_2_O–1.97MoO_3_–1.52SeO_2_–2.07Cr_2_O_3_
NCL3	20.03SiO_2_–3.79P_2_O_5_–32.52B_2_O_3_–4.97Na_2_O–5.23CaO–6.27K_2_O–2.69MgO–2.72Al_2_O_3_–3.20TiO_2_–10.67Fe_2_O_3_–0.40Li_2_O–2.05BaO–1CoO–2.43V_2_O_5_–2.03Cr_2_O_3_
NCL4	16.28SiO_2_–9.63P_2_O_5_–37.77B_2_O_3_–4.21Na_2_O–3.80CaO–6.38K_2_O–2.73MgO–5.52ZnO–7.03SrO–2.12CaF_2_–1.08CuO–1.95MoO_3_–1.51SeO_2_
NCL5	60.28P_2_O_5_–4.09Na_2_O–5.28CaO–5.32K_2_O–3.80MgO–3.84ZnO–4.10MnO_2_–9.77SrO–0.75TiO_2_–2.75Sb_2_O_3_
NCL6	4.61SiO_2_–68.19P_2_O_5_–6.68B_2_O_3_–4.76Na_2_O–5.38CaO–2.71K_2_O–1.55MgO–1.50CaF_2_–1.67MnO_2_–0.76CuO–0.72CoO–1.46Cr_2_O_3_
NCL7	39.96SiO_2_–9.46P_2_O_5_–12.39Na_2_O–11.19CaO–2.50K_2_O–1.61MgO–15.44AgO–2.13TiO_2_–4.26Fe_2_O_3_–1.06CuO
NCL8	38.93SiO_2_–10.24P_2_O_5_–2.01B_2_O_3_–8.94Na_2_O–12.11CaO–6.78K_2_O–2.91MgO–6.27MnO_2_–1.17ZnO–2.30Fe_2_O_3_–1.49SrO–2.81CaF_2_–0.57CuO–0.54CoO–1.04MoO_3_–0.80SeO_2_–1.10Cr_2_O_3_
AW	4.6 MgO–44.7–CaO–34SiO_2_–16.2P_2_O_5_–0.5 CaF_2_

Glass frits of all the compositions were ground in a one-bowl zirconia ball milling machine (Planetary Mono Mill Pulverisette 6, Fritsch GmbH, Germany) using a rotational speed of 400 rpm for 30 min, and then sieved using a mechanical sieve shaker (Impact Test Equipment Ltd., UK) to have a final particle size about 20 μm and below 53 μm.

Powders were prepared for pressing through mixing with an isopropanol solution (Sigma Aldrich, UK) in the proportion 1:3 (w/w). Powders were then pressed using an automatic hydraulic press (Specac-Atlas™ 8T, Specac Ltd., UK) to make 10 mm diameter and 2.5 mm high pellets. The pressed pellets were then sintered in a furnace (Carbolite 1200 CWF, Carbolite GmbH, Germany), with the sintering times and temperatures defined by the results of the hot stage microscopy analysis, reported in Section 2.3.2.

### 2.3 Physico-chemical characterisation

#### 2.3.1 Microstructural characterisation

Powder glasses and dense pellets were sputtered with a thin layer of gold (approximately 10 nm, sputter time 40 s at 40 mA), and afterward analysed using a Philips XL30 Field-Emission Environmental Scanning Electron Microscope (ESEM FEG), which is fitted with a Rontec Quantax system for the Energy-Dispersive Spectroscopy (EDS) analysis. All the images were taken at an operation voltage of 20 kV, and working distance between 5 and 10 mm.

#### 2.3.2 Hot stage microscopy (HSM)

The sintering ability of the novel glass powders was determined using hot stage microscopy (Misura®, Expert System Solutions, Italy). Tests were performed in air using a heating rate of 10 °C/min up to 1200 °C. Glass powders were manually pressed into a small cylindrical die (2 × 3 mm) and placed on a 10 × 15 × 1 mm alumina support. During the process the specimens were observed by a video camera and images of the changing sample profile were acquired up to 1450 °C. Afterwards, the sample shrinkage at different temperatures was calculated from the variation of the sample area, using the following formula:

$$\text{shrinkage}\;\left(\%\right)=\frac{A_{T}}{A_{0}}\times 100$$

where *A*_0_ (mm^2^) was the initial area of the specimen at room temperature and *A*_*T*_ (mm^2^) was the area of the specimen at the temperature *T*.

#### 2.3.3 XRD Analysis

To investigate the nature of the novel materials. XRD analysis was performed using a PANalytical X'Pert Pro MPD, powered by a Philips PW3040/60 X-ray generator, and fitted with an X'Celerator detector. Diffraction data was acquired by exposing powder samples to Cu-K_α_ X-ray radiation, at 40 kV and 40 mA. The data were collected over a 2θ range between 5 and 80°, with a step size equal to 0.0334°, a counting time per step of 200 s using the scanning X'Celerator detector. Phase identification was carried out using the PANalytical X'Pert HighScore Plus© software.

#### 2.3.4 Ion leaching evaluation

Un-sintered glass powders with a concentration of 10 mg/ml were immersed in deionised water (Veolia Water Technologies, UK) and incubated under an atmosphere of 5% CO_2_ and 95% air at 37 °C. After each storage period (1, 3, 7, 14 and 28 days), the specimens were removed *via* filtration, and filtrates retained to analyse the ion release potential of each compositions. An inductively coupled plasma optical emission spectroscopy (ICP-OES) (Specto-Ciros-Vision, Sheffield University, UK), which allows simultaneous multi-element analysis following the calibration of the instrument by standards of known concentrations of the elements of interest, was employed.

### 2.4 Biological characterisation

The *in vitro* cytotoxicity of the novel glass formulations was evaluated according to ISO 10993–5 [47] using rat calvaria osteoblast cells in indirect contact with glass powders up to 7 days. Each powder sample was firstly sterilised using 100% ethanol solution, and after added to Dulbecco's modified Eagle medium (DMEM, Gibco® UK) at three different concentrations (0.1, 1 and 10 mg/ml) and incubated for 24 h at 37 °C. After incubation, the glass-conditioned medium was filtered through a 0.22 μm microbiological filter, and used for *in vitro* tests.

Cells at early passages were provided by Institute of Cellular Medicine (Medical School, Newcastle University, UK), and then were cultured in T75 flasks at 37 °C in a humidified incubator with 5% CO_2_, using DMEM supplemented with 10% fetal bovine serum (FBS), 1% penicillin-streptomycin and 1% glutamine (Gibco®, UK). Cells were seeded at a density of 1x10^4^cells/well in 96-well plates and incubated at 37 °C. After 24 h culture period the culture media was discarded and replaced with the filtered solution for indirect cytotoxicity testing. Rat OBs cultured in the absence of glass powders were used as negative control.

The culture plates were then incubated for 1 and 7 days. The cytotoxic effect was measured exposing each well to 3-(4,5-dimethylthiazol-2-yl)-2,5-diphenyltetrazolium bromide (MTT, Sigma–Aldrich, UK) solubilisation at a concentration of 0.5 mg/ml. 100 μl of MTT solution was added to each well and incubated at 37 °C for another 4 h. MTT was taken up only by active cells and reduced in their mitochondria to insoluble purple formazan granules. The medium was then removed and 100 μl of dimethyl sulfoxide (DMSO) was added to dissolve the precipitated formazan. The absorbance of the solution was evaluated spectrophotometrically at a wavelength of 570 nm, and acquired using a Sunrise microplate reader (Tecan Group Ltd., Switzerland).

The absorbance values from three replicates were averaged and statistically analysed using two–way analysis of variance (ANOVA) followed by Bonferroni *post hoc* analysis. *P*-values < 0.05 were considered statistically significant.

## 3 Results

### 3.1 Glass production and powder processing

During synthesis, composition NCL5 could not form a glass at any temperature up to 1500 °C, which was the highest temperature the available furnaces could reach. Glass frits from the seven compositions which did form a glass, were ground using a zirconia ball mill, and sieved to obtain fine powder in the range 0-53 μm.

### 3.2 Glass powder microstructural analysis

**Fig. 1:**
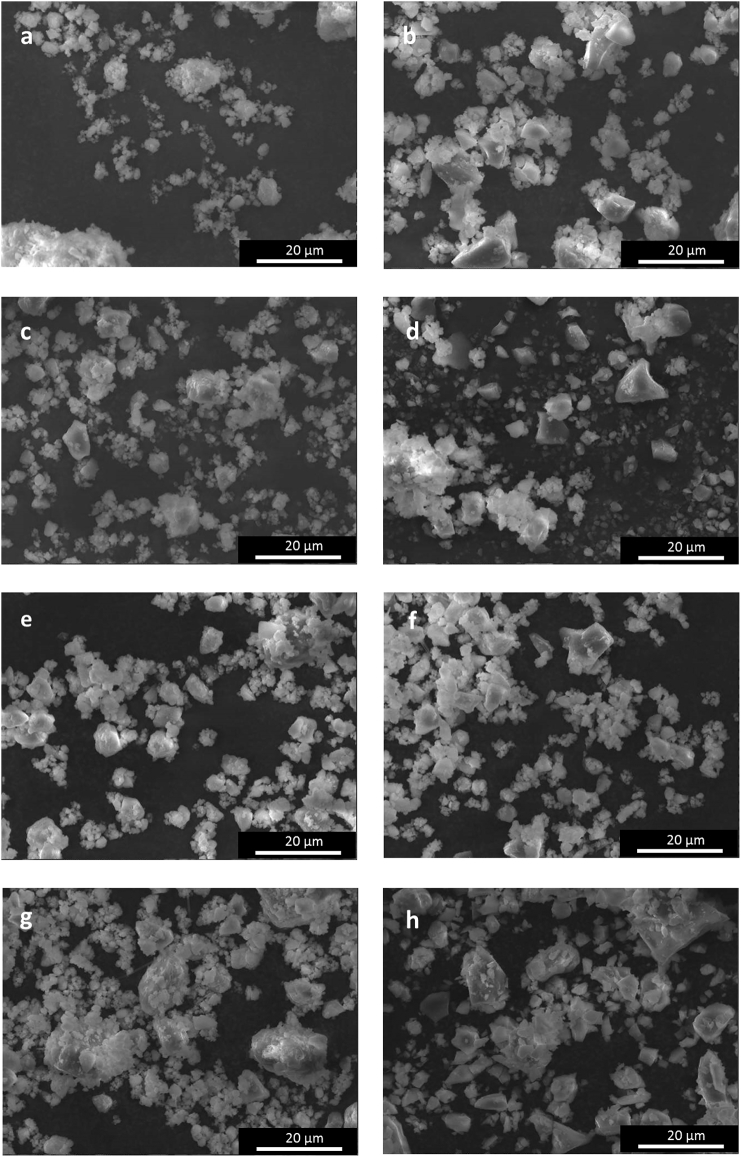
SEM analysis (magnification 1500 ×) showing the glass powders morphology: a) NLC1, b) NCL2, c) NCL3, d) NCL4, e) NCL6, f) NCL7, g) NCL8, and h) AW.

Fig. 1(a–h) shows the morphology of as-synthesised glass powders. Non-spherical and irregular shape particles with sharp edges can be observed for all the compositions. Furthermore, it can be seen that for all the glasses most of the particles were very fine (ranging from 20 μm to 53 μm), with the presence also of grains smaller than 10 μm, which tended to compact producing aggregates.

### 3.3 HSM

**Fig. 2:**
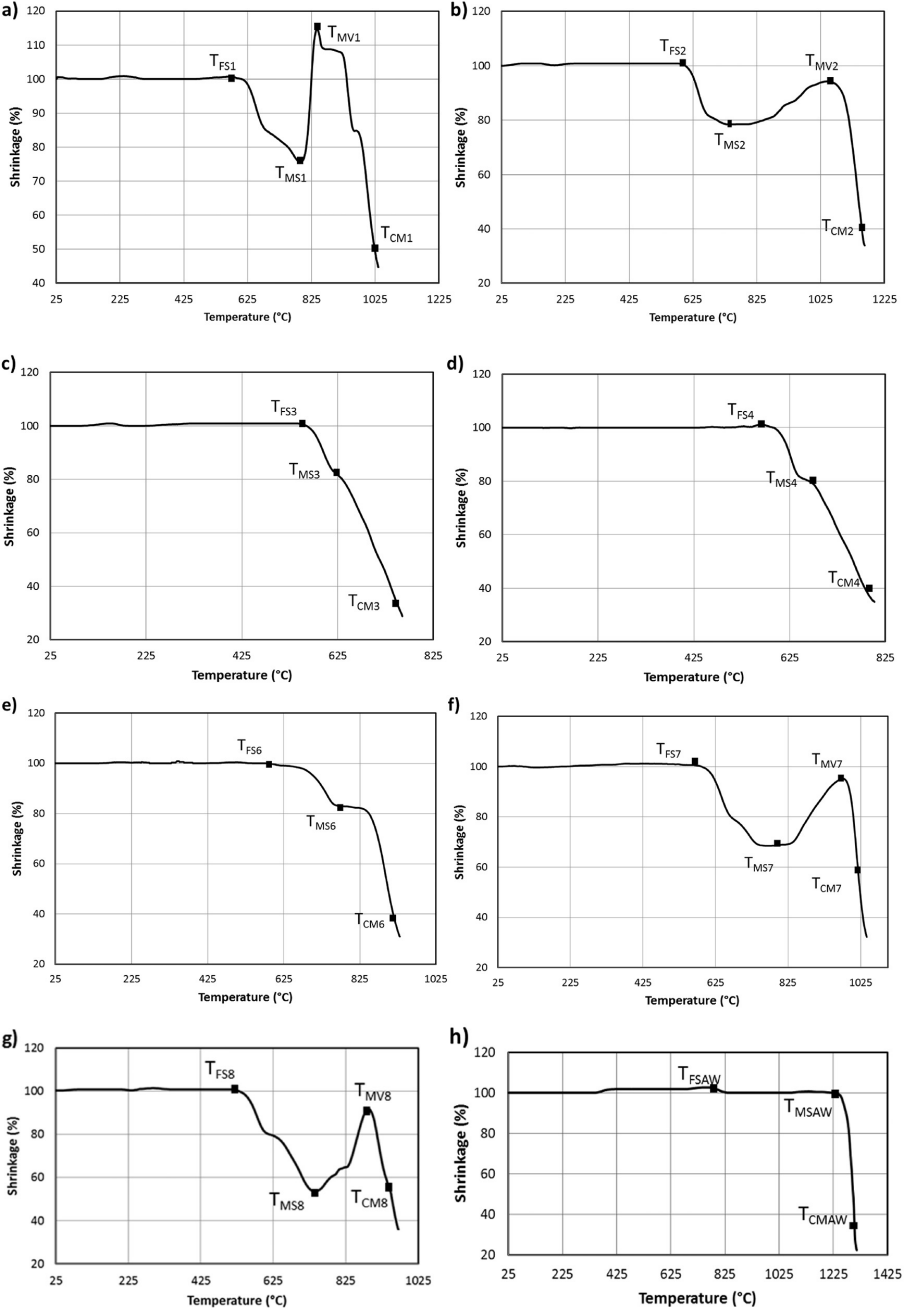
Shrinkage profile derived from hot stage microscopy as function of temperature for: a) NCL1, b) NCL2, c) NCL3, d) NCL4, e) NCL6, f) NCL7, g) NCL8 and h) AW.

The experiments started at room temperature (T_R_) with heating rate of 10 °C/min up to 1450 °C. All the specimens maintained their initial rectangular shape before the first shrinkage temperature (T_FS_), which varied between 550 °C and 1225 °C (see Fig. 2(a–h)). For temperatures higher than the corresponding T_FS_, the samples started to shrink until the temperature of maximum shrinkage (T_MS_). The variation of sample dimensions during the sintering process are reported in Fig. 2(a–h). Fig. 2(a–b, f–g) reveal also how the silicate-based specimens (NCL1, NCL2, NCL7 and NCL8), before reaching the melting status at complete melting temperature (T_CM_), started to expand up to their temperature of maximum volume (T_MV_). Furthermore, these silicate-based glasses showed a similar thermal profile, whereas the phosphate-based (NCL6) had a thermal curve (Fig. 2(e)) more comparable to the borate-based glasses (NCL3 and NCL4) (Fig. 2(c–d)).

### 3.4 XRD

**Fig. 3:**
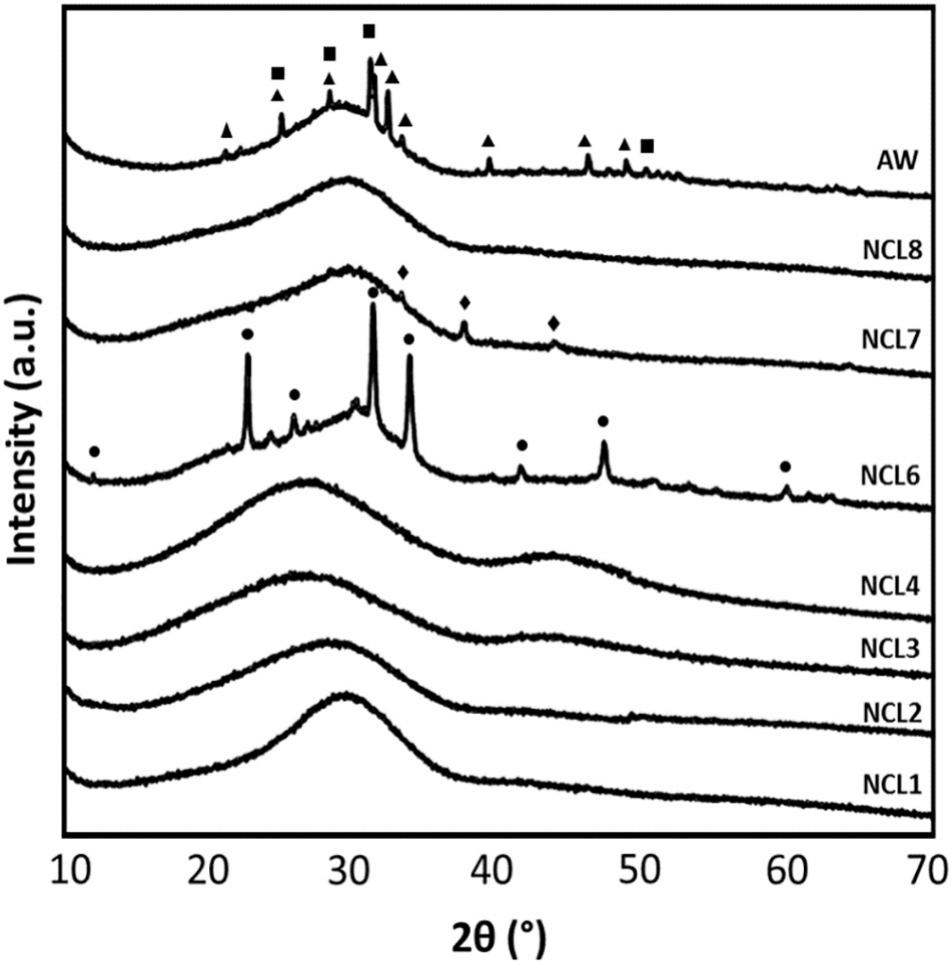
XRD patterns of as-synthesised glass powders (▲ hydroxylapatite, ■ β-wollastonite, ◆ silver ● calcium sodium phosphate).

The XRD patterns of the raw materials are reported in Fig. 3. For NCL1, NCL2, NCL3, NCL4, and NCL8 the presence of a broad peak, common for glass samples, indicated the completely amorphous nature of these compositions. The amorphous peak was detected at 2θ values between 25° and 30°, and confirmed that NCL1, NCL2, NCL3, NCL4 and NCL8 formulations were free from any detectable crystalline phase. Different patterns were detected for NCL6 formulation, which showed a glass-ceramic nature with a crystalline phase identified as calcium sodium phosphate (ICDD ref. code 01-074-1950). Moreover, for the NCL7 composition a silver crystalline phase (ICDD ref. code 04-003-1425) was detected. Regarding the AW composition, the presence of hydroxylapatite phase (ICDD ref. code 01-080-6260) and a less intense β-wollastonite phase (ICDD ref. code 04-010-0710) were observed, proving the glass-ceramic structure of this material.

### 3.5 Ion leaching evaluation

**Fig. 4:**
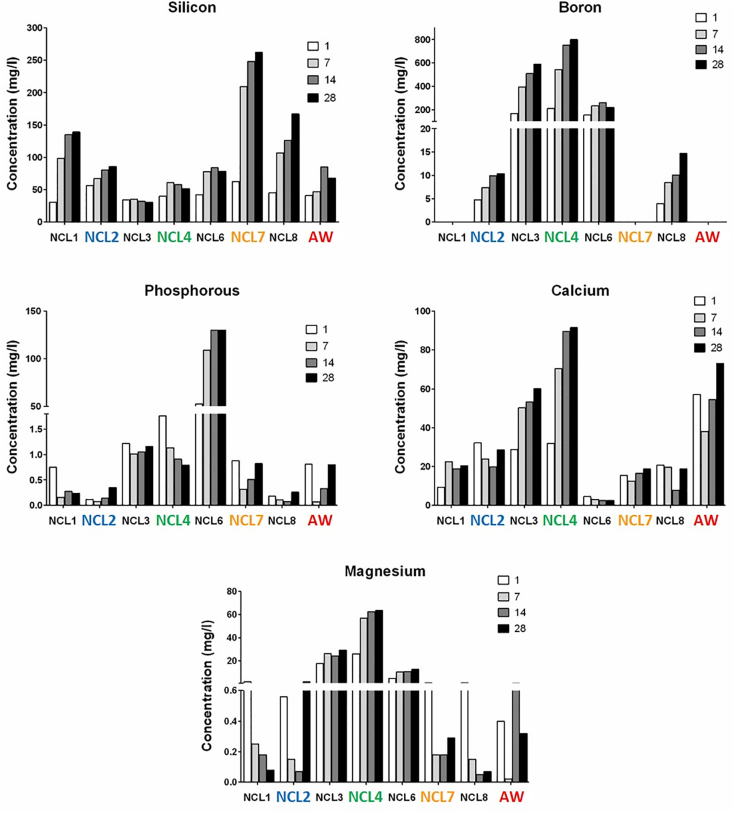
Ionic concentrations of Si, P, B, Ca and Mg released into deionised water from all the formulations, without refreshing the solutions and at different time points (1, 7, 14 and 28 days).

The ionic release of the common elements (Si, P, B, Ca and Mg) of each glass composition is reported in Fig. 4. It is interesting to observe that, except for NCL2 composition, the amount of silicon released in solution after 28 days was proportional to the molar content present in the main formulation. As network former, boron was released very quickly in comparison to silicon and phosphorous, and it displayed a progressive increase over the time period for both NCL3 and NCL4 compositions. Moreover, it was found that the amount of phosphorous, as main network former, in the NCL6 composition was released more slowly than boron from NCL3 and NCL4 formulations.

### 3.6 Bioceramic pellets sintering and characterisation

**Fig. 5:**
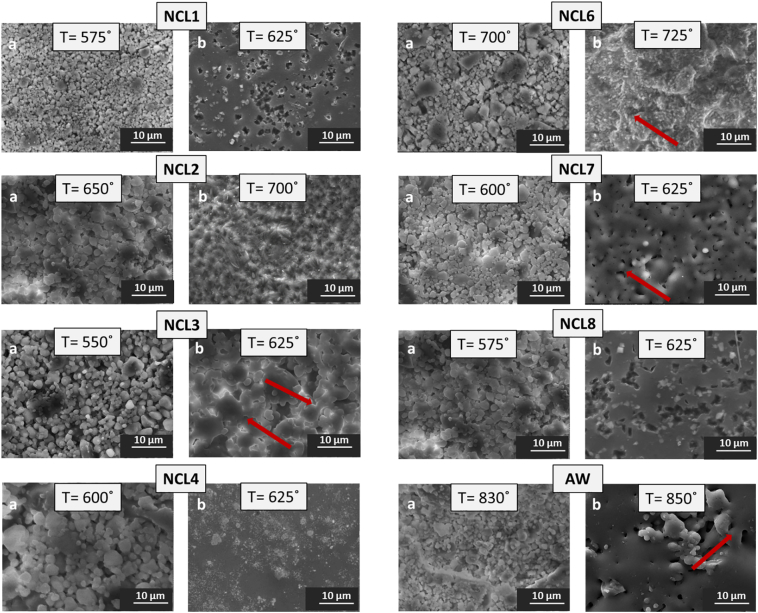
SEM micrographs of bioceramic pellet surface at (a) low and (b) appropriate sintering level (temperature = °C, magnification = 2500 ×); red arrows indicate necking formation phenomena. (For interpretation of the references to colour in this figure legend, the reader is referred to the web version of this article.)

Sintering was performed with a heating rate of 10 °C/min up to a maximum temperature in the range 550–850 °C, which was held for 1 h; after this the samples were left to cool down at room temperature. Sintering temperatures were selected on the basis of the HSM results. SEM investigations were conducted to evaluate the effect of different maximum temperatures on sample morphology. Fig. 5 reports the pellet surface of each composition for two heat treatments per material. Micrographs (a) of Fig. 5 show a poor sintering level for all the formulations, where glass particles started to aggregate, but were not properly sintered. The microstructures reported in micrographs (b) of Fig. 5 demonstrate how an increase in the heating temperatures led to an appropriate densification status, as a result of increased liquid phase. Particularly for NCL1, NCL3, NCL6, NCL7, NCL8 and AW compositions the formation of sintering necks (red arrows in the figure) became evident.

In light of the SEM microstructural observations, and after a thorough qualitative analysis, a summary of the heating temperatures that qualitatively were considered to provide the best densification degree are shown in Table 3.

**Table 3:** Sintering intervals of the novel glass formulations (derived from HSM) and optimal sintering temperatures of dense bioceramic pellets.

Code	Composition	HSM sintering interval (°C)	Sintering temperature (°C)
NCL1	Silicate-based	575–785	625
NCL2	Silicate-based	600–730	700
NCL3	Borate-based	555–625	625
NCL4	Borate-based	550–650	625
NCL6	Phosphate-based	580–775	725
NCL7	Silicate-based	575–785	625
NCL8	Silicate-based	500–730	625
AW	Silicate-based	800–1225	850

### 3.7 Biocompatibility assay

**Fig. 6:**
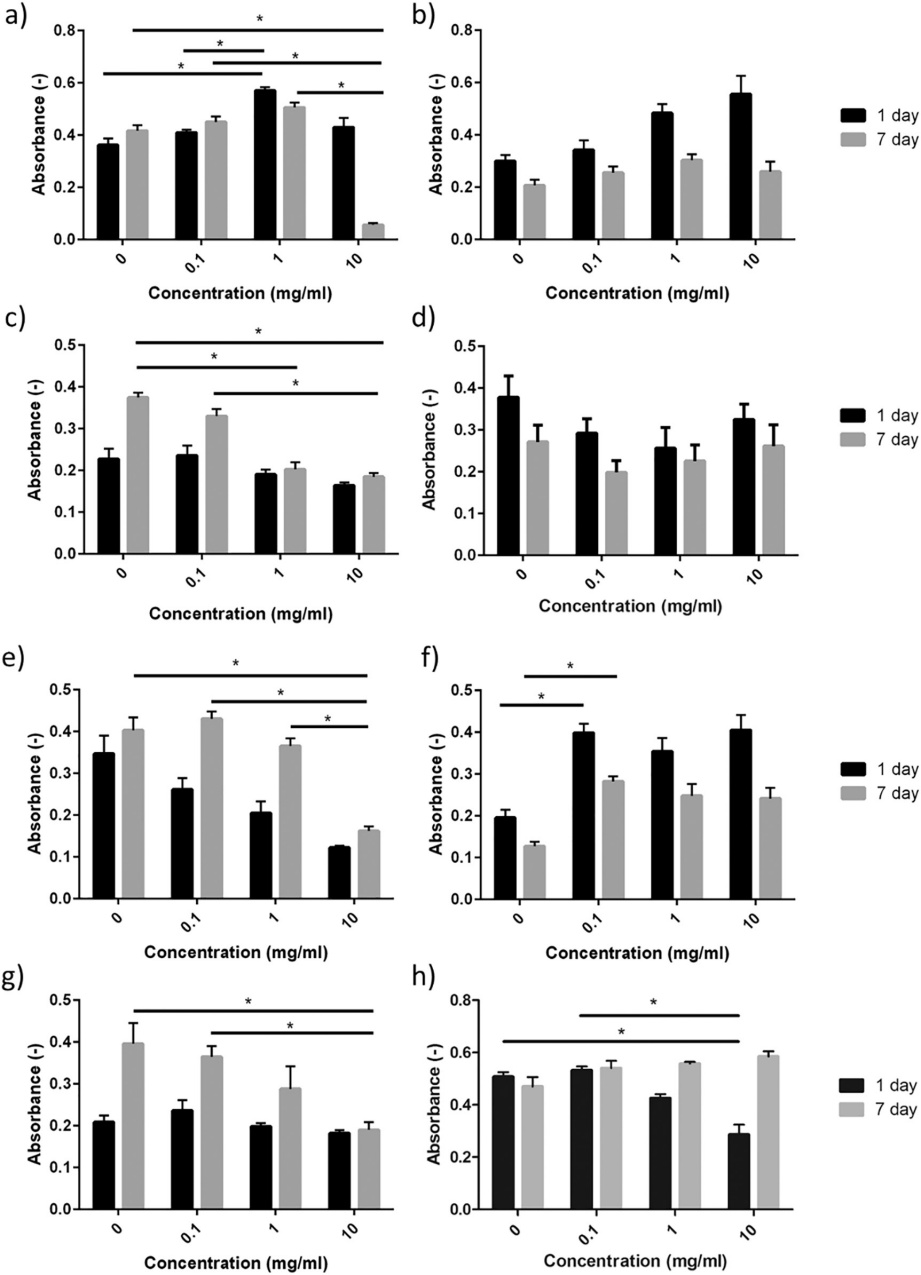
Effect of a) NCL1, b) NCL2, c) NCL3, d) NCL4, e) NCL6, f) NCL7, g) NCL8 and h) AW glass powders (measured in triplicate) on formazan formation after indirect contact with rat osteoblast cells, evaluated through MTT assay after 1 day and 7 days in culture. Error bars represent the standard deviation (* = *P* < 0.05).

The MTT results are summarised in Fig. 6. For NCL1 the cells displayed good metabolic activity after 1 day in culture for all concentrations tested, however the effect of the glass became detrimental after 7 days in culture at a concentration of 10 mg/ml. NCL2 shows not meaningful positive effects on metabolic activity after day 1, with no significant negative effect compared to control at day 7. The NCL3, NCL6 and NCL8 formulations all show cytotoxic effects on cell mitochondrial activity after 7 days in culture, which increases with increasing quantities of glass powder. NCL4 shows a broadly uniform response over the time period, with no significant differences in response across concentrations or time points. The NCL7 composition showed a positive effect on cell viability and proliferation at both time points, being this statistically significant for the lowest concentration after 7 days in culture. For AW glass-ceramic, the findings of this study confirmed the results of previous work [48], according to which the material initially has a slightly cytotoxic effect on cell mitochondrial activity, followed by enhanced cell proliferation.

## 4 Discussion

For this study eight novel bioceramic formulations were designed in order to develop biomaterials with beneficial performance towards bone tissue repair and regeneration, by using *i*) silicon dioxide, *ii*) phosphorous pentoxide and *iii*) boron trioxide as network formers.

The glasses were synthetized *via* a melting-quenching route; however, during the glass production their forming ability was composition dependant. Even though both NCL5 and NCL6 are phosphate-based glasses, the NCL5 formulation did not form a liquid at 1500 °C. This contradicts the commonly held view that phosphate–based glasses can be prepared at relatively low temperatures [32]. Based on the findings reported by Abou Neel et al., the incorporation of high density oxides like SrO_2_ in the phosphate glass structure might be associated with an increase of the glass melting temperature; and this is considered the most likely reason for the poor processability of the NCL5 formulation [49].

HSM analysis revealed that the silicate-based glasses (NCL1, NCL2, NCL7 and NCL8) displayed similar thermal profiles, characterised by an increase in sample dimensions after the maximum shrinkage temperature and before the melting onset occurred. These results are comparable with the findings reported by Baino et al., who found that a less complex silicate-based glass (CEL2) exhibited a significant volumetric expansion after the first densification step [14]. Within the scope of the formulations reported in Table 2, we conclude that the silicate glass sintering profiles are relatively insensitive to the presence of different network modifiers in the glass structure.

HSM analysis performed on NCL6 phosphate-based glass showed a thermal profile with densification that takes place in two steps (elbow-shape profile). Furthermore, similarly to ICEL2 phosphate glass (45% P_2_O_5_, 3% SiO_2_, 26% CaO, 7% MgO, 15% Na_2_O and 4% K_2_O) [50], NCL6 formulation displayed an HSM thermograph with a volume increase at temperatures higher than T_MS_. This observation is consistent with the findings reported by Arstila et al., according to which the elbow shape curve could indicate the possible sintering interval of the glass [51], [52]. Moreover, the presence of network modifiers such as MnO_2_, CaF_2_, CuO, CoO, Cr_2_O_3_, and in particular B_2_O_3_ could have affected the thermal behaviour of this formulation, leading to a shrinkage profile more comparable to the one developed by the borate-based compositions.

The sintering behaviour of borate-based bioglasses has to date been explored in less depth than silicate or phosphate based glasses. In this study, the two borate-based glasses (NCL3 and NCL4) exhibited similar one-step densification behaviour, with a small sintering interval in comparison to the silicate and phosphate-based glasses [53].

The selection of sintering temperatures is a key step during the manufacturing process to consolidate ceramic-based structures [54], [55]. Although a further optimisation process (based on thermal treatment and morphological analysis evaluation) was required, the outcomes from the HSM were a very useful guide in predicting the optimal sintering temperatures of bioceramic pellets: the temperatures that led to optimal consolidated structures, as demonstrated by SEM investigation, were all in the range of sintering temperatures provided by HSM [56].

According to the ICP-OES results, and by comparing the release of the main network formers, boron was released very quickly in comparison to silicon and phosphorus, therefore proving the highly reactive nature of borate-based glasses [5]. These findings reflect the study design (Table 1), which aimed to develop compositions with tailored degradation rates. It is also notable that the presence of boron in the glass structure, even as intermediate oxide, enhanced the release of the other elements (*i.e.* phosphorous release from NCL3 glass with respect to NCL1 composition) in the formulation.

For all the novel glasses, apart from the NCL2 composition, silicon leaching proved to be proportional to its molar content in the parental glass. Additionally, it was found that the release of phosphorus increased with soaking time only for the NCL6 formulation, in which it was used as network former, most likely as a result of the higher levels of phosphorous in NCL6 [32], [57].

The results of the *in vitro* cytotoxicity tests revealed that for high concentrations of glass powder (10 mg/l), cell mitochondrial activity was significantly reduced for NCL1, NCL3, NCL6 and NCL8 compositions [58], meaning that the NCL2, NCL4 and NCL7 formulations are suitable for further study. No single effect provided a direct correlation between the cytotoxicity and ionic release potential, but a number of observations can be made:

• For both the NCL1 and NCL3 formulations, it is most likely that the release of vanadium had a negative effect on cell mitochondrial activity [59], [60].
• The NCL6 composition did not contain many dopants, and because the release of Co, Cu and Cr after 28 days in immersion was quite low (data not shown) [61], it is most likely that the high levels of phosphorus and boron were the cause of the detrimental effect of this glass.
• No clear reasons for the toxicity of NCL8 were found; hence, the negative effect is most likely due to the combination of the different oxides.
• The highly reactive nature of the NCL4 borate-based glass enabled the release of strontium at a higher rate than NCL1 composition, which has been demonstrated to increase proliferation as well as differentiation of osteoblast cells [62], [63].
• The positive effect of the NCL7 formulation might derive from the combined effect of titanium, iron and copper, which enhance osteoblasts proliferation and activity [64].

## 5 Conclusion

This paper has presented a systematic process for the evaluation of novel bioceramic materials, considering basic glass processability, hot stage microscopy, ICP analysis and a biocompatibility assay. The main conclusions which can be drawn are:

(i) It is possible for phosphate-based glasses to have high melting temperatures, as the NCL5 formulation did not form a liquid at 1500 °C.
(ii) The sintering range identified by HSM consistently included the optimum sintering temperature, indicating the usefulness of this technique in defining sintering routines.
(iii) No direct correlation between ion release and cytotoxicity could be observed across all of the glasses studied, although silicon release seems to be mostly dependent on silicon content; and vanadium does not seem to have enhanced biocompatibility at the concentrations studied in this paper.
(iv) Of the eight proposed glasses the NCL2, NCL4 and NCL7 formulations have shown sufficient biocompatability to merit further study as new biomaterials.
